# A Method of Using a Pelvic C-Clamp for Intraoperative Reduction of a Zone 3 Sacral Fracture

**DOI:** 10.1155/2016/8586517

**Published:** 2016-12-22

**Authors:** Daniel H. Wiznia, Nishwant Swami, Chang-Yeon Kim, Michael P. Leslie

**Affiliations:** ^1^Department of Orthopaedics and Rehabilitation, Yale University School of Medicine, 800 Howard Avenue, New Haven, CT 06510, USA; ^2^Yale University, 800 Howard Ave., New Haven, CT, USA

## Abstract

It is challenging to properly reduce pelvic ring injuries that involve a zone 3 sacral fracture. Several open and closed reduction methods have been described. Percutaneous reductions are challenging, and improper reductions can have poor long-term outcomes. The pelvic C-clamp is a tool designed to provide emergency stabilization to patients suffering from c-type pelvic ring injuries. We describe a case in which a patient's open book pelvic ring injury with a zone three sacral fracture is reduced intraoperatively with the use of a pelvic C-clamp and stabilized with transsacral screws.

## 1. Introduction

Zone three sacral fractures are challenging to reduce, and they frequently require open reduction. Open treatment methodologies suffer from the common complication of wound breakdown. Indirect reduction with the use of percutaneous transsacral screws has been shown to be an effective method to fix unstable pelvic ring injuries with lower wound complications [1].

In an emergency setting, the pelvic C-clamp stabilizes pelvic ring injuries and controls hemorrhage [2]. With an assembly time of approximately ten minutes, the C-clamp staunches bleeding by applying compression indirectly to fracture surfaces and the veins of the presacral plexus [2–4]. Instruments similar to the C-clamp can be traced to Germany from reports published in the 1960s. The C-clamp in its current form came into wider use after reports were published by Ganz et al. in 1991 and Buckle et al. in 1994 [3, 5]. In contrast to other tools available for compression of pelvic ring injuries, the C-clamp allows for unrestricted access to the abdomen, pelvis, and proximal femur. These properties make the C-clamp ideal to be used intraoperatively to obtain compression of the posterior pelvic ring.

In this paper, we describe a patient who suffered an open book pelvic ring injury with a zone 3 sacral fracture. We describe a surgical technique in which the pelvic C-clamp was used nontraditionally intraoperatively as a reduction modality with the patient in the prone position in conjunction with percutaneous transsacral screw fixation. We have obtained the written informed consent of the patient for print and electronic publication, as well as permission for the use of radiographs. Potential conflicts of interest do not exist for any of the authors.

## 2. Case Report

**Figure 1:**
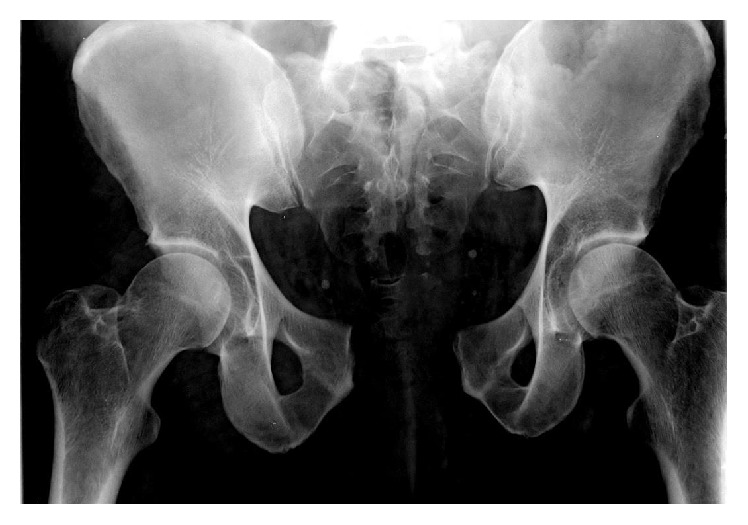
Presenting pelvis injury X-ray.

**Figure 2:**
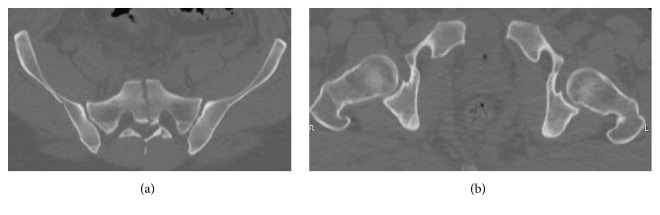
Presenting injury CT scan axial cuts ((a) and (b)).

A 44-year-old man suffered a pelvic ring injury and a comminuted proximal humerus fracture-dislocation in a motorcycle collision. The patient was transferred from an outside hospital where he had been intubated and placed in a pelvic binder and fluid resuscitated. At the time of presentation at our facility, the patient was deemed hemodynamically stable with a GCS of 11. A pelvic hematoma could be seen on the outside hospital CT. The pelvic ring injury consisted of a pubic symphysis disruption and a markedly displaced complete zone 3 sacral fracture from S1 through the coccyx (Figures 1 and 2). On exam, he was noted to have left lower extremity weakness but intact sensation.

On hospital day 3, the patient was brought to the operating room for fixation of the anterior and posterior pelvic ring injuries. First, he underwent open reduction and internal fixation of his anterior pelvic ring injury. While the patient was positioned supine, the pubic symphysis was reduced with a Farabeuf clamp and stabilized with a 6-hole symphyseal plate with 3.5 mm screw fixation. The posterior ring was then compressed with S1 and S2 transsacral screws compressing the zone 3 sacral fracture.

**Figure 3:**
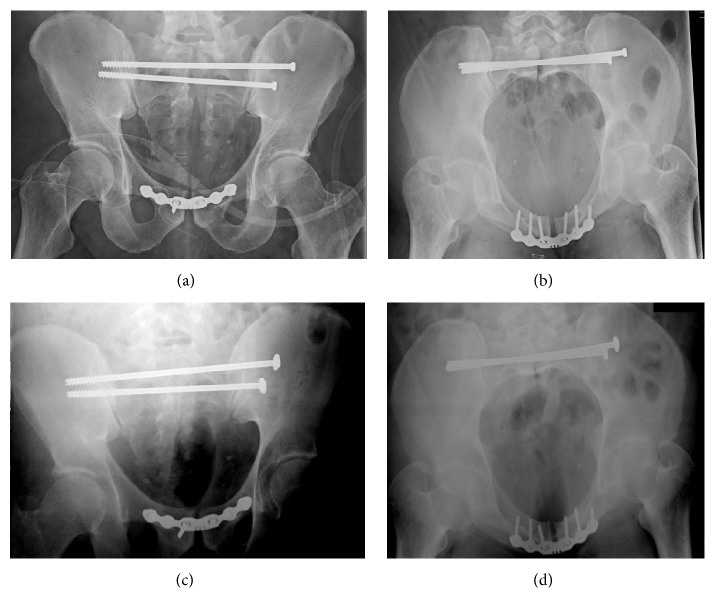
Pelvis (a) anteroposterior and (b) inlet views before using C-clamp and pelvis (c) anteroposterior and (d) inlet views after using C-clamp.

On hospital day 4, postoperative radiographs demonstrated unacceptable residual displacement of his sacrum through the zone 3 fracture site (Figures 3(a) and 3(b)). This displacement of the sacrum was thought to be secondary to tension banding of the anterior pelvic ring preventing the posterior ring reduction. The patient returned to the operating room on hospital day 12 for revision in the prone position. Transsacral guide wires were placed through the S1 and S2 transsacral cannulated screws. Both screws were removed. A pelvic C-clamp was then applied over the S2 transsacral wire. Radiographs demonstrated an anatomic reduction. With the C-clamp holding the sacral fracture reduction, S1 transsacral screw was engaged into the far ilium allowing for more compression across the fracture site. An additional guide wire was passed at the S2 level for further stability. Another transsacral screw was placed across the fracture utilizing one of the S2 guide wires (Figures 3(c) and 3(d)).

The patient's hospital course was complicated by pneumonia and a deep vein thrombosis. The patient was discharged to short term rehabilitation on hospital day 30, with restrictions not to bear more than 25 pounds to the left lower extremity. At the patient's 8-week follow-up appointment, he complained of burning and numbness over the buttocks. At the patient's three-month follow-up, he complained of sacral and bilateral buttock pain, as well as sexual dysfunction. At a recent 3-year follow-up the patient is weight bearing as tolerated without restriction. He has returned to motorcycling.

## 3. Discussion

To our knowledge, no published case describes the use of a pelvic C-clamp in a nonemergency setting for the stabilization and reduction of a zone 3 sacral fracture. In addition, very little has been described in terms of using the pelvic C-clamp in nonemergent settings to achieve intraoperative reduction of pelvic ring injuries.

The following paragraph provides a historical perspective regarding the C-clamp. Pneumatic antishock garments (PASG) and medical antishock trousers (MAST) were used in the 1960s and 1970s to stabilize patients with severe pelvic trauma and hemorrhage [6, 7]. To improve upon the limited abdominal access of these methods as well as to provide improved stability to posteriorly unstable fractures, Mohanty et al. designed the sliding bar C-clamp and Buckle et al. designed the curved arm ratchet gear pelvic stabilizer [7, 8]. The C-clamp was found to provide significant compression to the posterior ring [9], allowed access to the abdomen, and could be retained as a temporary measure while definitive fixation was achieved [5, 10, 11]. The use of the pelvic C-clamp has decreased as C-clamp related complications have become more recognized and with the routine use of pelvic binders at the scene of the injury [11]. The C-clamp is currently manufactured and sold by DePuy Synthes Trauma division, West Chester, Pennsylvania [12].

It should be noted that the current version of the DePuy Synthes C-clamp is cannulated, which allows the C-clamp cannulated nails to be placed over a guidewire. This feature allowed the application of the C-clamp over the guide wire inserted through the previously placed S2 screw.

The patient in our case report suffered a pelvic ring injury that consisted of a pubic symphysis disruption and a complete zone 3 sacral fracture. The patient's pelvic ring injury can be classified as an anterior-posterior compression type 3 pelvic ring injury. The complete zone 3 sacral fracture acts as though there has been disruption of the anterior and posterior sacroiliac ligaments. This rare type of anterior-posterior compression pelvic ring injury is described by Bellabarba et al. in a case series of 10 patients. The pelvis is externally rotationally displaced and is vertically stable, with unilateral, partial disruption of the posterior arch [13].

In their case series of 10 patients who suffered open book pelvis injuries with zone 3 sacral fractures, Bellabarba et al. describe eight patients treated with open reduction and internal fixation. Regarding patient outcomes, Bellabarba et al. described three out of ten patients who complained of sexual dysfunction. We observed this complication in our patient. Bellabarba et al. also describe one patient who required a suprapubic catheter because of a urethral stricture. None of the patients in the series suffered motor or sensory neurologic compromise [13].

We attribute the challenge to reduce this patient's sacrum as secondary to his supine positioning and tension banding of the anterior ring. In addition, the patient's obesity (BMI 37) made the reduction challenging. During the second operation, by positioning the patient in the prone position, the patient's weight may have acted to provide a compressive force to the posterior ring, and the C-clamp provided additional compression across the fracture.

It should be noted that while the surgical team's use of intraoperative fluoroscopy ensured a safe path for the SI screws during the primary procedure, the malreduction was not identified, likely because the quality of the intraoperative fluoroscopy was deteriorated by the patient's obesity. A more critical review by the surgical team may have been able to identify the malreduction intraoperatively and provided an opportunity to revise the reduction.

**Figure 4:**
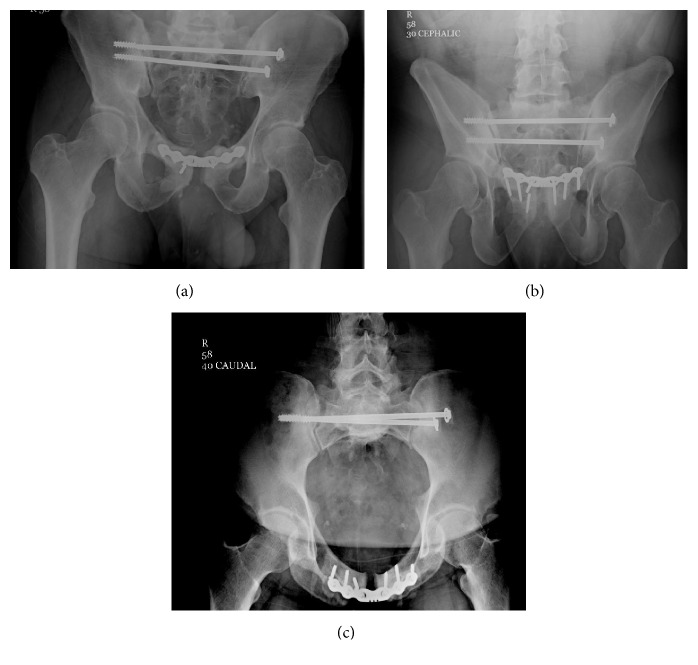
(a) Pelvis AP, (b) outlet, and (c) inlet views at two-year follow-up.

Figure 3 demonstrates the degree of the malreduction after the first surgery (Figures 3(a) and 3(b)) and the degree in which the diastasis was compressed after the second surgery (Figures 3(c) and 3(d)). In biomechanical studies, C-clamps have been shown to apply some of the highest compressive loads on the posterior pelvic ring [14]. Figures 4(a), 4(b), and 4(c) demonstrate the healed pelvis with multiple broken screws two years postoperatively.

There are two studies which describe the use of the C-clamp to reduce Denis zone 2 sacral fractures. Wright et al. describe successful reduction and fixation of a sacroiliac joint dissociation associated with a contralateral Denis classification zone 2 sacral fracture in a 26-year-old male using the pelvic C-clamp. The authors describe the use of the C-clamp intraoperatively, which allowed for reduction of the dissociated sacroiliac joint and the placement of transsacroiliac screws [15]. Wright et al. recommend conducting the surgery with the patient in the supine position, obtaining fixation of the anterior pelvic ring injury first, and applying the C-clamp at the S2 level [15]. Quintero et al. describe using the pelvic C-clamp to reduce a Denis classification zone 2 sacral fracture with approximately 5 cm lateral displacement. Their technique describes the intraoperative use of the pelvic C-clamp to obtain definitive fixation with the patient positioned prone [16].

As a note of caution, those who decide to use the C-clamp should be properly trained in its application. There are true risks in incorrectly inserting the pins such as breaching the iliac wing, the sciatic notch, and the hip joint [17].

In conclusion, the use of the pelvic C-clamp can be considered for patients who have suffered a posterior pelvic ring disruption in which there is a large amount of diastasis without vertical instability. Patient positioning must be considered preoperatively given how critical it can be to the success of reduction maneuvers.
